# CT-based radiomics for predicting Ki-67 expression in lung cancer: a systematic review and meta-analysis

**DOI:** 10.3389/fonc.2024.1329801

**Published:** 2024-02-07

**Authors:** Xinmin Luo, Renying Zheng, Jiao Zhang, Juan He, Wei Luo, Zhi Jiang, Qiang Li

**Affiliations:** ^1^ Department of Radiology, People’s Hospital of Yuechi County, Guang’an, Sichuan, China; ^2^ Department of Oncology, People’s Hospital of Yuechi County, Guang’an, Sichuan, China; ^3^ Department of Radiology, Yuechi County Traditional Chinese Medicine Hospital in Sichuan Province, Guang’an, Sichuan, China

**Keywords:** radiomics, CT-scan, artificial intelligence, Ki-67, lung cancer, machine learning

## Abstract

**Background:**

Radiomics, an emerging field, presents a promising avenue for the accurate prediction of biomarkers in different solid cancers. Lung cancer remains a significant global health challenge, contributing substantially to cancer-related mortality. Accurate assessment of Ki-67, a marker reflecting cellular proliferation, is crucial for evaluating tumor aggressiveness and treatment responsiveness, particularly in non-small cell lung cancer (NSCLC).

**Methods:**

A systematic review and meta-analysis conducted following the preferred reporting items for systematic review and meta-analysis of diagnostic test accuracy studies (PRISMA-DTA) guidelines. Two authors independently conducted a literature search until September 23, 2023, in PubMed, Embase, and Web of Science. The focus was on identifying radiomics studies that predict Ki-67 expression in lung cancer. We evaluated quality using both Quality Assessment of Diagnostic Accuracy Studies (QUADAS-2) and the Radiomics Quality Score (RQS) tools. For statistical analysis in the meta-analysis, we used STATA 14.2 to assess sensitivity, specificity, heterogeneity, and diagnostic values.

**Results:**

Ten retrospective studies were pooled in the meta-analysis. The findings demonstrated that the use of computed tomography (CT) scan-based radiomics for predicting Ki-67 expression in lung cancer exhibited encouraging diagnostic performance. Pooled sensitivity, specificity, and area under the curve (AUC) in training cohorts were 0.78, 0.81, and 0.85, respectively. In validation cohorts, these values were 0.78, 0.70, and 0.81. Quality assessment using QUADAS-2 and RQS indicated generally acceptable study quality. Heterogeneity in training cohorts, attributed to factors like contrast-enhanced CT scans and specific Ki-67 thresholds, was observed. Notably, publication bias was detected in the training cohort, indicating that positive results are more likely to be published than non-significant or negative results. Thus, journals are encouraged to publish negative results as well.

**Conclusion:**

In summary, CT-based radiomics exhibit promise in predicting Ki-67 expression in lung cancer. While the results suggest potential clinical utility, additional research efforts should concentrate on enhancing diagnostic accuracy. This could pave the way for the integration of radiomics methods as a less invasive alternative to current procedures like biopsy and surgery in the assessment of Ki-67 expression.

## 1 Introduction

Lung cancer is a major global health challenge, leading in cancer-related deaths and posing a significant threat to public health. Despite notable progress in diagnosis and therapy, it remains a persistent global health burden (1, 2). Non-small cell lung cancer (NSCLC) takes precedence, constituting 85% of total cases and involving adenocarcinoma and squamous cell carcinoma (3–5). Disturbingly, more than two-thirds of NSCLC instances receive a diagnosis at an advanced stage (6, 7). Therefore, early diagnosis of this cancer is very crucial for its management.

Ki-67, a marker reflecting cellular proliferation, provides crucial information about the tumor’s aggressiveness and its potential responsiveness to treatment (8). Its prediction is crucial in lung cancer due to its role as a proliferation marker. Its significance lies in assessing tumor cell proliferation, aiding prognostic evaluations and treatment decisions in NSCLC (9). Ki-67 has emerged as a prognostic marker associated with overall survival (OS) and disease-free survival (DFS) in NSCLC. Higher Ki-67 expression indicates poorer outcomes, suggesting its potential to predict disease aggressiveness and guide personalized treatment approaches (10, 11).

In the realm of lung cancer, the significance of imaging has been revitalized, particularly in the context of baseline staging and response assessment. The emergence of cutting-edge technologies like artificial intelligence (AI) has further elevated the role of imaging, transforming it into a potent biomarker for noninvasive tumor characterization (12, 13). This resurgence underscores the potential of advanced imaging methods, which are empowered by computational advancements, to provide comprehensive insights into Ki-67 levels. Such noninvasive approaches promise to enhance our understanding of tumor characteristics, obviating the necessity for invasive procedures and opening new avenues for precise diagnostic and prognostic assessments (14).

Radiomics is an emerging field within medical imaging that aims to extract extensive quantitative data from routine medical images, such as those obtained from CT, MRI, and positron emission tomography (PET) (15, 16). The process typically involves identifying and segmenting a region of interest (ROI), which can be done manually or using automated algorithms (17). From these segmented regions, high-dimensional features are extracted, falling into two main categories: semantic features, which describe morphological aspects of lesions, and agnostic features, which are mathematical (18, 19). Functioning in diverse capacities such as tumor classification, survival prediction, and therapy response assessment, radiomic signatures are pivotal in crafting imaging biomarkers for personalized therapy (12, 20). The interdisciplinary realm of radiogenomics seamlessly intertwines imaging with genomics and molecular data. Despite grappling with methodological challenges, radiomics persistently holds promise, offering nuanced insights beyond the confines of traditional cancer evaluation methods (21).

A meta-analysis on predicting EGFR mutation in NSCLC revealed that AI-based algorithms, utilizing radiomics features, serve as valuable and noninvasive tools for predicting EGFR mutation status, with excellent diagnostic accuracy (22). Recently, many meta-analyses on radiomics-based methods have been published to investigate the overall diagnostic performance of the available studies in the field. This will help in obtaining a standpoint regarding the current radiomics methods for predicting biomarkers in cancers (23–25). Thus, this study aims to provide a meta-analysis of the radiomics studies for predicting Ki-67 expression in lung cancer for the first time and evaluate their quality as well.

## 2 Materials and methods

This systematic review and meta-analysis was conducted according to the preferred reporting items for systematic review and meta-analysis of diagnostic test accuracy studies guidelines (PRISMA-DTA) (26). No review protocol was registered.

### 2.1 Literature search

Two authors independently conducted a thorough literature search of the PubMed, Embase, and Web of Science databases to find papers that used radiomics for Ki-67 prediction in lung cancer and that were published up until September 23, 2023. Following terms were used in search: (Ki-67) AND (Lung Cancer) AND (Radiomics). The retrieved references were exported to the Mendeley Reference Manager. The detailed search method is shown in **Supplementary Table S1**.

### 2.2 Eligibility criteria

The inclusion criteria were as follows based on the PICO questions (population, intervention, comparison, and outcomes): (P) patients with lung cancer, (I) radiomics methods were applied to identify Ki-67 expression in lung cancer, (C) diagnosis was made by histopathological examination (preferably via surgery), and (O) providing sufficient data for constructing 2×2 table including true positive (TP), false positive (FP), false negative (FN), and true negative (TN) values for evaluating sensitivity and specificity. The exclusion criteria were as follows: (a) article published not in the English language, (b) case reports, reviews, letters, meetings, abstracts, comments, and guidelines, (c) articles with insufficient data for constructing 2×2 tables (d) articles without radiomic analysis, (e) cohort overlaps, and (f) studies that expression of Ki-67 was not predicted. The primary outcome was the prediction of Ki-67 using radiomics by providing sensitivity and specificity. Secondary outcome measures included area under the curve (AUC), diagnostic odds ratio (DOR), and positive and negative likelihood ratios (PLR, NLR). After the titles and abstracts were examined by two different reviewers, the entire texts were evaluated to see if they qualified for inclusion. If there were disagreements amongst the reviewers, they were resolved by discussion or, if required, consultation with a third reviewer.

### 2.3 Data extraction

The basic data of the included studies were extracted using a data extraction table. The data that was extracted included: first author name, publication year, study design (retrospective vs. prospective), country, imaging modality (e.g., CT or PET), population (case and controls), age of patients, cut-off for Ki-67 in immunohistochemistry (IHC) staining, number of extracted features, ROI structure (3D vs. 2D), number of features (selected/extracted), name of the software for feature extraction, type of radiomics features, feature reduction algorithm, and algorithm for model construction. For meta-analysis, these data were extracted as well: TP, FN, TN, and FP. Upon evaluating the diagnostic efficacy of multiple algorithms on an identical sample, the algorithm yielding the most favorable categorization outcomes was selected.

### 2.4 Quality assessment

Two tools, including QUADAS-2 and the RQS scoring system, were used for quality assessment. QUADAS-2 is a tool used for assessing the quality of diagnostic accuracy studies in systematic reviews. QUADAS-2 provides a structured framework for evaluating the risk of bias and concerns regarding the applicability of diagnostic accuracy studies. It focuses on four key domains: patient selection, index test, reference standard, and flow and timing. The tool is widely used in evidence-based medicine to ensure rigorous evaluation of the quality of diagnostic studies included in systematic reviews (27). The RQS is another system for measuring the quality of radiomics studies with sixteen components with a maximum point of 36 points (28). Two independent reviewers conducted the quality assessment, and any disagreements were resolved by discussion.

### 2.5 Statistical analysis

The meta-analysis was carried out in STATA 14.2 using “midas” module. A coupled forest plot was generated to depict the pooled sensitivity and specificity of the radiomics studies. Cochran’s Q and Higgins’ I^2^ were computed to assess the heterogeneity among the studies included in this meta-analysis. I^2^ values ranging from 0 to 25%, 25 to 50%, 50 to 75%, and > 75% indicate very low, low, medium, and high heterogeneity, respectively. Pooling studies and effect size were evaluated using a random-effects model, emphasizing the consideration of heterogeneity when estimating the distribution of true effects across studies. The hierarchical summary receiver operating characteristic (HSROC) model was used to produce the summary receiver operating characteristic (SROC) curve and estimate the pooled AUC. Other diagnostic values, including DOR, PLR, and NLR were pooled. Spearman’s rank correlation test was used to investigate the threshold effect. Meta-regression was used to investigate the possible source of heterogeneity based on different subgrouping. When publication bias was present, following excluding each study one by one, sensitivity analysis was conducted to evaluate the stability of the pooled values. Leave-one-out analysis was performed using OpenMeta[Analyst] software. A Deek’s funnel plot was generated to show publication bias. All p-values lesser than 0.05 were considered significant.

## 3 Results

### 3.1 Literature search

**Figure 1:**
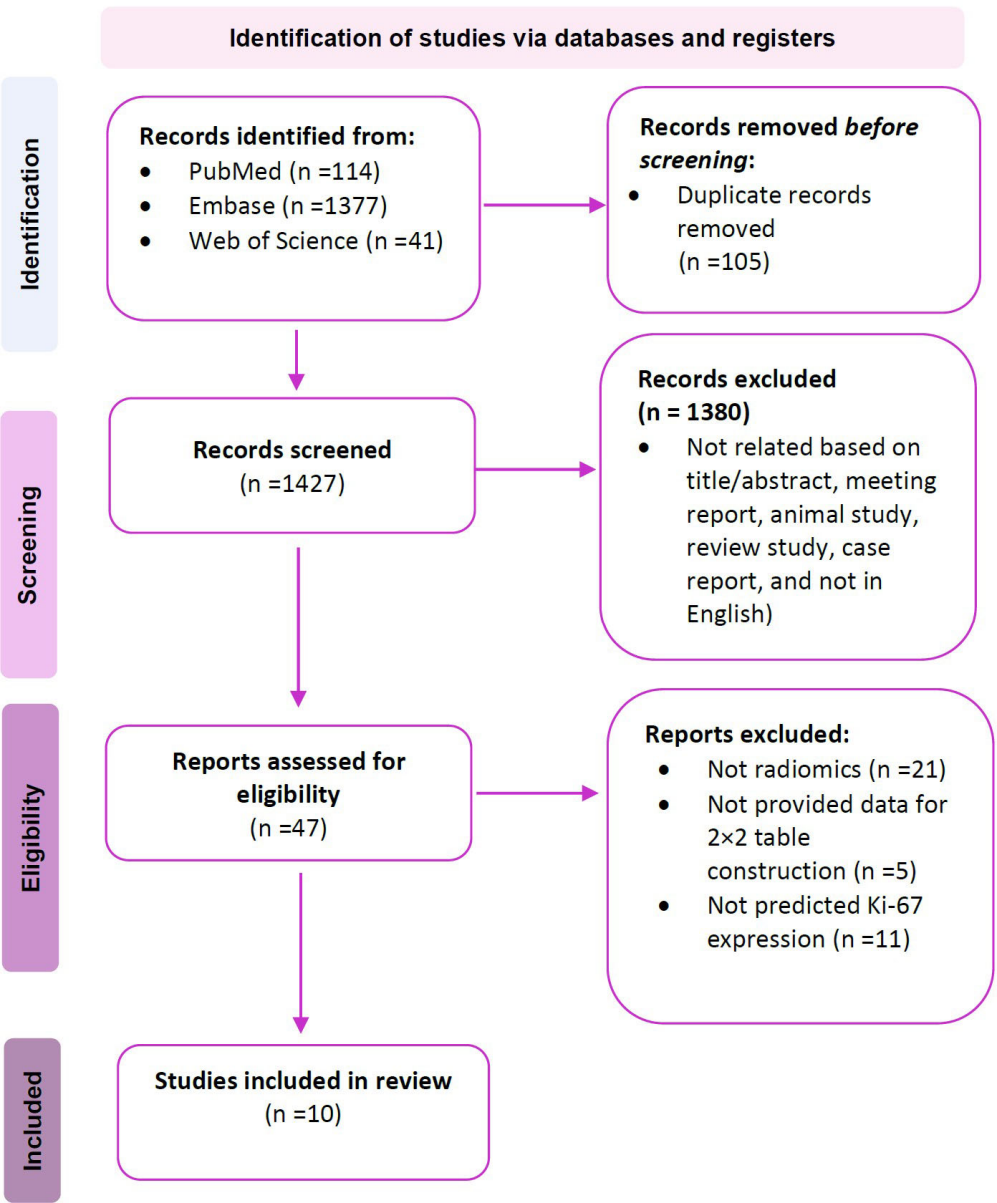
PRISMA flowchart of the study.

As per the search methodology outlined in the methods section, a total of 1,532 were identified from different databases. Following the removal of 105 duplicate records, 1,427 titles were subjected to evaluation. During the title and abstract assessment, 1,380 citations were excluded due to not meeting inclusion criteria (e.g., lack of relevance based on title/abstract, inclusion of meeting reports, reviews, case reports, and not being in the English language). After careful revision, an additional 37 articles were excluded: 21 studies were found to not involve radiomics, 11 did not predict Ki-67 expression, and 5 did not provide sufficient data for constructing a 2×2 table. This resulted in the final inclusion of 10 articles for the meta-analysis (29–38). The study flow chart has been depicted in **Figure 1**.

### 3.2 Characteristics of the included studies

All of the included studies were designed retrospectively, conducted in China, and used CT scans as the only imaging modality. Seven studies were conducted at single-center institutions (29, 30, 33–36, 38), while three studies adopted a multicenter approach (31, 32, 37). Three studies used contrast-enhanced CT scans for their images (30, 33, 35). Different threshold values were utilized to designate Ki-67 expression as positive, from 5% to 50%, with 40% emerging as the most frequently applied threshold (30, 31, 33, 35) compared to others. In nine studies, segmentation involved the use of 3D ROI, while one study did not specify the segmentation structure (36). Manual ROI segmentation was employed in eight studies (29, 31–34, 36–38), whereas in two studies, the segmentation process was semiautomatic (30, 35). Extracted features ranged from 105 to 3362. The extracted features exhibited a wide range, spanning from 105 (30, 33) to 3362 (35). Shape-based features were the most commonly extracted features in nine studies (29–37). Feature extraction software were different, including 3D slicer (n=3) (30, 33, 34), AK software (n=3) (29, 31, 37), PyRadiomics (n=2) (32, 35), Feature Explorer (n=1) (36), and MaZda (n=1) (38). The least absolute shrinkage and selection operator (LASSO) was the most frequently used feature reduction algorithm (n=6) (29, 31, 34–37). Logistic regression (LR) was the most frequently used algorithm for building radiomics signature (n=8) (29–33, 35–37). The characteristics of the included studies are shown in **Table 1**.

**Table 1:** Characteristics of the included studies.

Author and year	Center	Study design	Number of lesions/patients	Age	Ki-67 cut-off	Imaging modality	ROI (3D vs. 2D)	ROI segmentation	Features type	Selected features(n)/Extracted features(n	Feature extraction software	Feature reduction algorithm	Modeling algorithm
**Zhou et al. (** 30 **),**	One	Rertro	110	62 (36–77)	40%	CT	3D	Semiautomatic	Shape, GLCM, and GLSZM	12/105	3D slicer	Backward elimination method	LR
**Gu et al. (** 38 **),**	One	Rertro	245	59 (31–85) years	50%	CT	3D	Manual	Greyscale histogram features, absolute gradients, and co-occurrence matrix	20/245	MaZda	Random forest feature selection algorithm (RFFS)	L2-LOG, LDA, CART, KNN, SVM, and **RF**.
**Huang et al. (** 29 **),**	One	Rertro	237/215	56 (22-82)	5%	CT	3D	Manual	First order, shape, GLCM, GLRLM, GLSZM, GLDM, and NGTDM.	8/1316	AK software	ANOVA, RFE, and LASSO	LR
**Fu et al. (** 33 **),**	One	Rertro	110/110	62 (36–77)	40%	CT	3D	Manual	Shape, GLDM, GLCM, first order, GLRLM, GLSZM, NGTDM	12/105	3D slicer	Chi‐square or Fisher’s exact tests	LR
**Yan et al. (** 37 **),**	Two	Rertro	153/153	56.2 ± 11.0 (26–79)	5%	CT	3D	Manual	first order, shape, GLCM, GLRLM, and NGTDM	20/1,316	AK software	mRMR and LASSO	LR
**Zhu et al. (** 34 **),**	One	Rertro	769/769	55.07 ± 9.36	10%	CT	3D	Manual	Shape-related, first order, texture, wavelet, and Laplacian of Gaussian (LoG)	13/2446	3D slicer	LASSO	SVM
**Bao et al. (** 36 **),**	One	Rertro	206/206	59.16 ± 9.99 (27-80)	10%	CT	–	Manual	first order, shape, GLCM, GLRLM, GLSZM, GLDM, and NGTDM	30/1316	Feature Explorer	mRMR and LASSO	LR
**Dong et al. (** 32 **),**	Four	Rertro	132	58.8 (27–80)	14%	CT	3D	Manual	Shape-based features, first-order features, texture features, and transform features	38/1,287	PyRadiomics	Mann-Whitney U test, Spearman test, (RF) based Boruta algorithm	LR
**Liu et al. (** 31 **),**	Five	Rertro	211/211	–	40%	CT	3D	Manual	first-order statistical features, shape features, texture features, and higher-order features.	5/1316	AK software	mRMR and LASSO	LR
**Sun et al. (** 35 **),**	One	Rertro	137/137	64.01 ± 10.55; (17–85)	40%	CT	3D	Semiautomatic	Shape, texture, first order, and wavelet features	14/3362	PyRadiomics based	ICC, variance thresholding, SelectKBest and LASSO	LR, **SVM**, and DT

### 3.3 Quality assessment

#### 3.3.1 QUADAS-2

**Figure 2:**
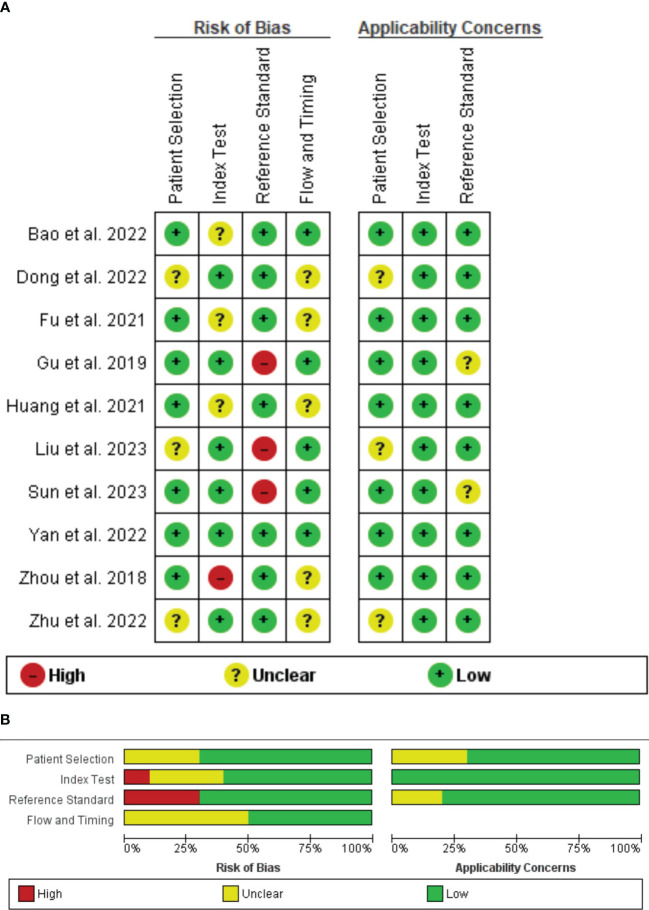
QUADAS quality assessment per study **(A)** and per domain **(B)**.

The result of the quality assessment based on the QUADAS-2 tool showed that in the patient selection domain, there were unclear risks of bias and unclear applicability concerns for three studies due to not specifying inclusion/exclusion criteria (31, 32, 34). For the index test, there was an unclear risk of bias for three studies due to not using cross-validation methods for modeling (29, 33, 36). However, as they matched research questions, no applicability concern was detected. In reference standard domain, three studies were detected as having a high risk of bias due to using biopsy for obtaining specimens (31, 35, 38). Finally, there was an unclear risk of bias for five studies due to not mentioning the interval between imaging acquisition and histopathological examination (29, 30, 32–34). Therefore, the quality of the included studies was almost acceptable (**Figure 2**).

#### 3.3.2 RQS

The ten studies obtained an average RQS score of 10.7, with individual scores ranging from 1 to 16 out of 36 points. The average score was 29%, and the study with the highest rating achieved 44%. Nearly half of the studies fell within the score range of 10 to 16. Only two studies employed imaging at multiple points (33, 35). None of the studies used phantom study prospective design, cost-effectiveness analysis, comparison to the gold standard, and open science items from the RQS checklist. Two studies received -5 points in validation items due to not using validation cohorts (30, 38). Image protocol quality, multiple segmentation, feature reduction, and biological correlation analysis were complete in all studies. **Table 2** represents the RQS scores for each study and item.

**Table 2:** RQS quality assessment of the included studies.

Study	I_1_	I_2_	I_3_	I_4_	I_5_	I_6_	I_7_	I_8_	I_9_	I_10_	I_11_	I_12_	I_13_	I_14_	I_15_	I_16_	RQS
Zhou et al. (30),	1	1	0	0	1	0	1	1	1	0	0	-5	0	0	0	0	1
Gu et al. (38),	1	1	0	0	1	1	1	1	2	0	0	-5	0	0	0	0	3
Huang et al. (29),	1	1	0	0	1	0	1	1	1	1	0	2	0	0	0	0	9
Fu et al. (33),	1	1	0	1	1	1	1	1	2	1	0	2	0	2	0	0	14
Yan et al. (37),	1	1	0	0	1	1	1	0	2	2	0	4	0	2	0	0	15
Zhu et al. (34),	1	1	0	0	1	1	1	0	2	0	0	2	0	0	0	0	9
Bao et al. (36),	1	1	0	0	1	1	1	0	1	1	0	2	0	2	0	0	11
Dong et al. (32),	1	1	0	0	1	1	1	1	2	1	0	5	0	2	0	0	16
Liu et al. (31),	1	1	0	0	1	1	1	0	2	1	0	5	0	2	0	0	15
Sun et al. (35),	1	1	0	1	1	1	1	0	2	2	0	2	0	2	0	0	14
Mean Score	1	1	0	0.2	1	0.8	1	0.5	1.7	0.9	0	1.4	0	1.2	0	0	10.7

I1, Image Protocol Quality; I2, Multiple Segmentation; I3, Phantom Study; I4, Imaging at Multiple Points; I5, Feature Reduction; I6, Multivariable Analyses; I7, Biological Correlation; I8, Cut-off Analyses; I9, Discrimination Statics; I10, Calibration Statics; I11, Prospective Study; I12, Validation; I13, Comparison to Gold Standard; I14, Potential Clinical Application; I15, Cost Effectiveness Analyses; I16. Open science and Data.

### 3.4 Meta-analysis

#### 3.4.1 Diagnostic performance in training cohorts

**Figure 3:**
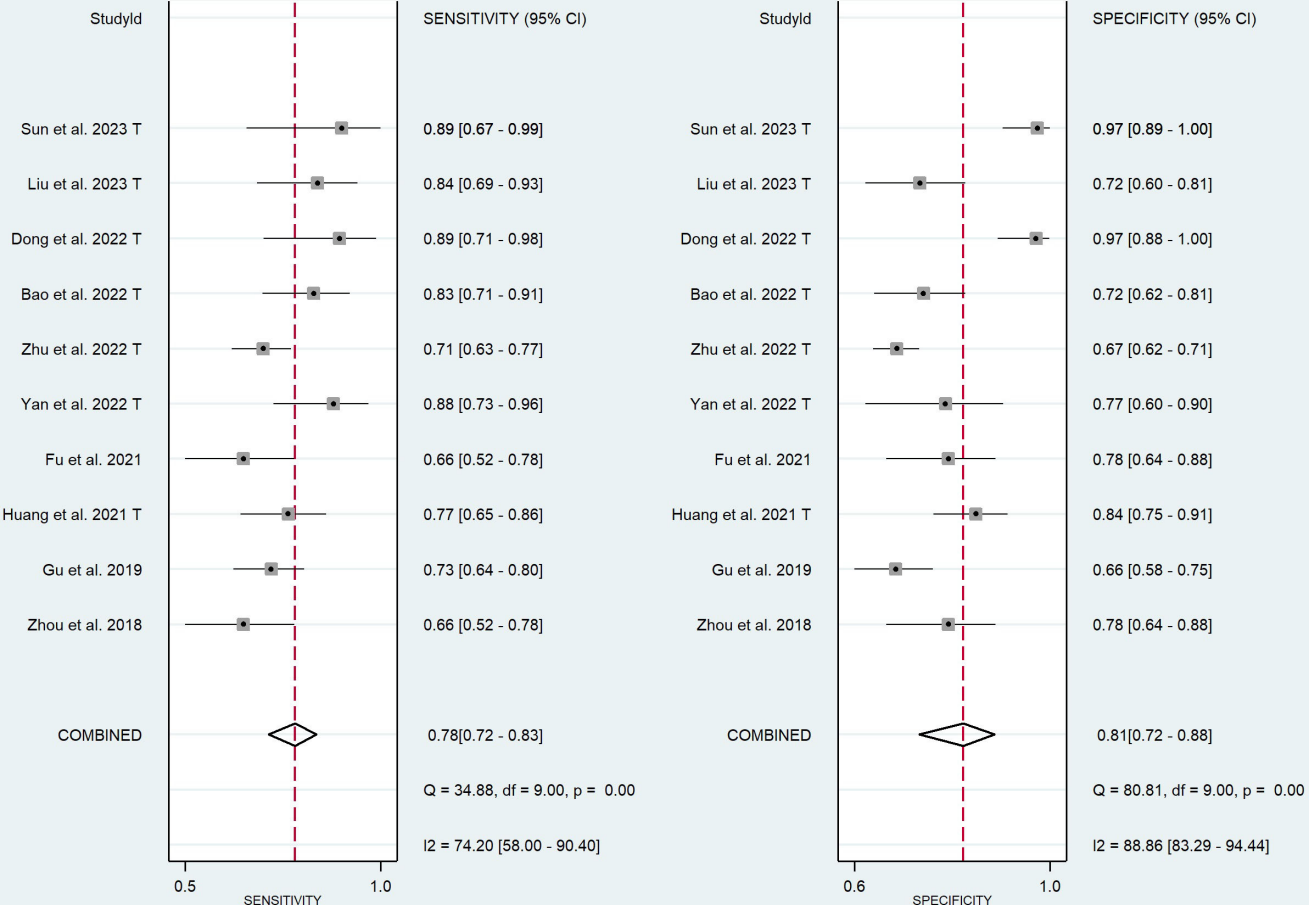
Coupled forest plot of the diagnostic performance in training cohorts.

**Figure 4:**
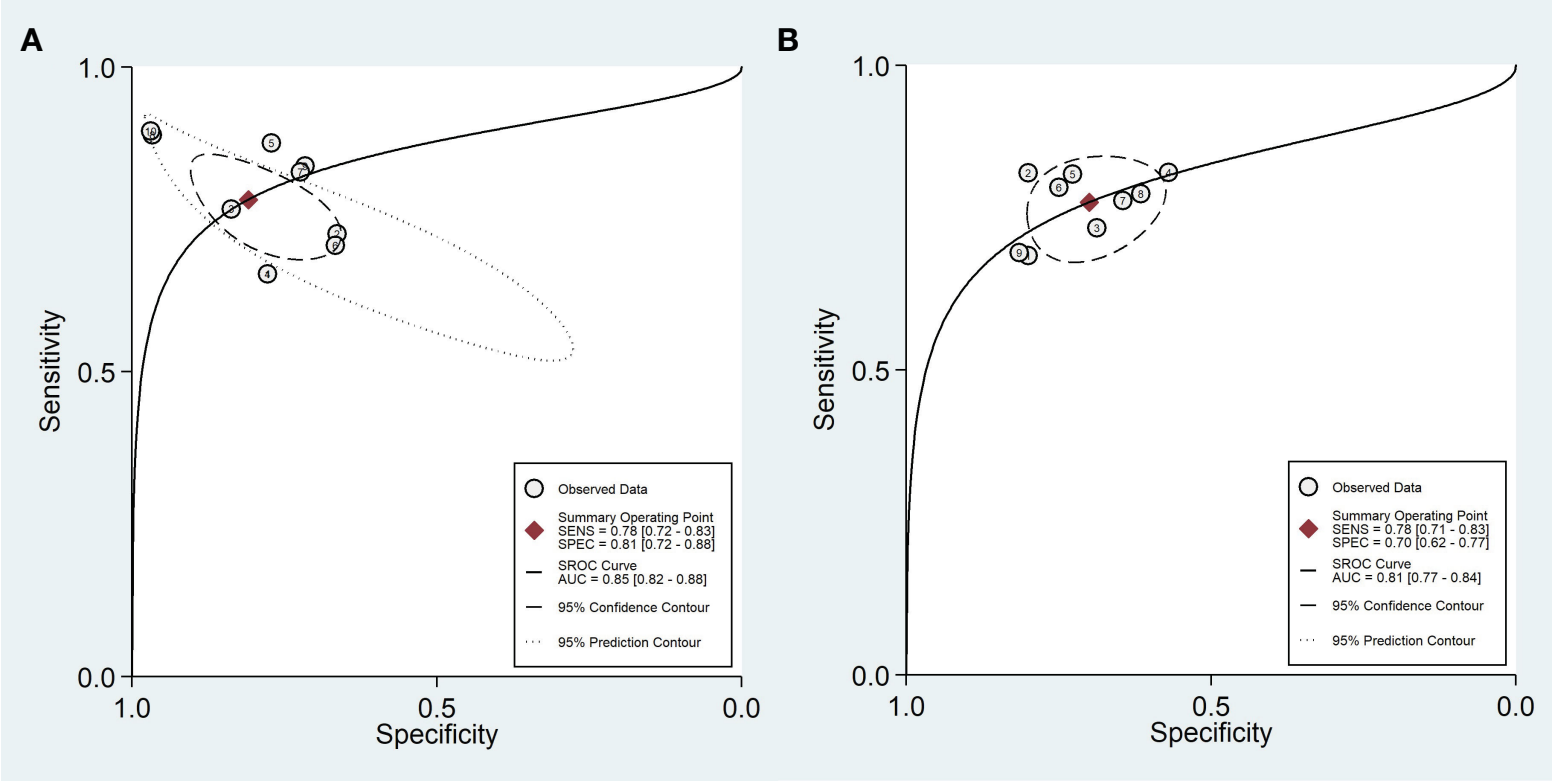
Summary ROC curve with confidence and prediction regions in training **(A)** and validation **(B)** cohorts.

Ten training cohorts consisting of 660 lesions with high and 1016 lesions with low Ki-67 were included for meta-analysis. The pooled sensitivity, specificity, DOR, PLR, NLR, and AUC with 95% confidence interval (CI) were 0.78 (0.72-0.83), 0.81 (0.72-0.88), 15 (7-41), 4.1 (2.6-6.6), 0.27 (0.19-0.37), and 0.85 (0.82-0.88), respectively. The coupled forest plot showing sensitivity, specificity, and heterogeneity indicators (I^2^ and Cochran’s Q) for training cohorts is depicted in **Figure 3**. In addition, the summary ROC curve (SROC) with pooled AUC value of the training cohorts is depicted in **Figure 4A**.

#### 3.4.2 Diagnostic performance in validation cohorts

**Figure 5:**
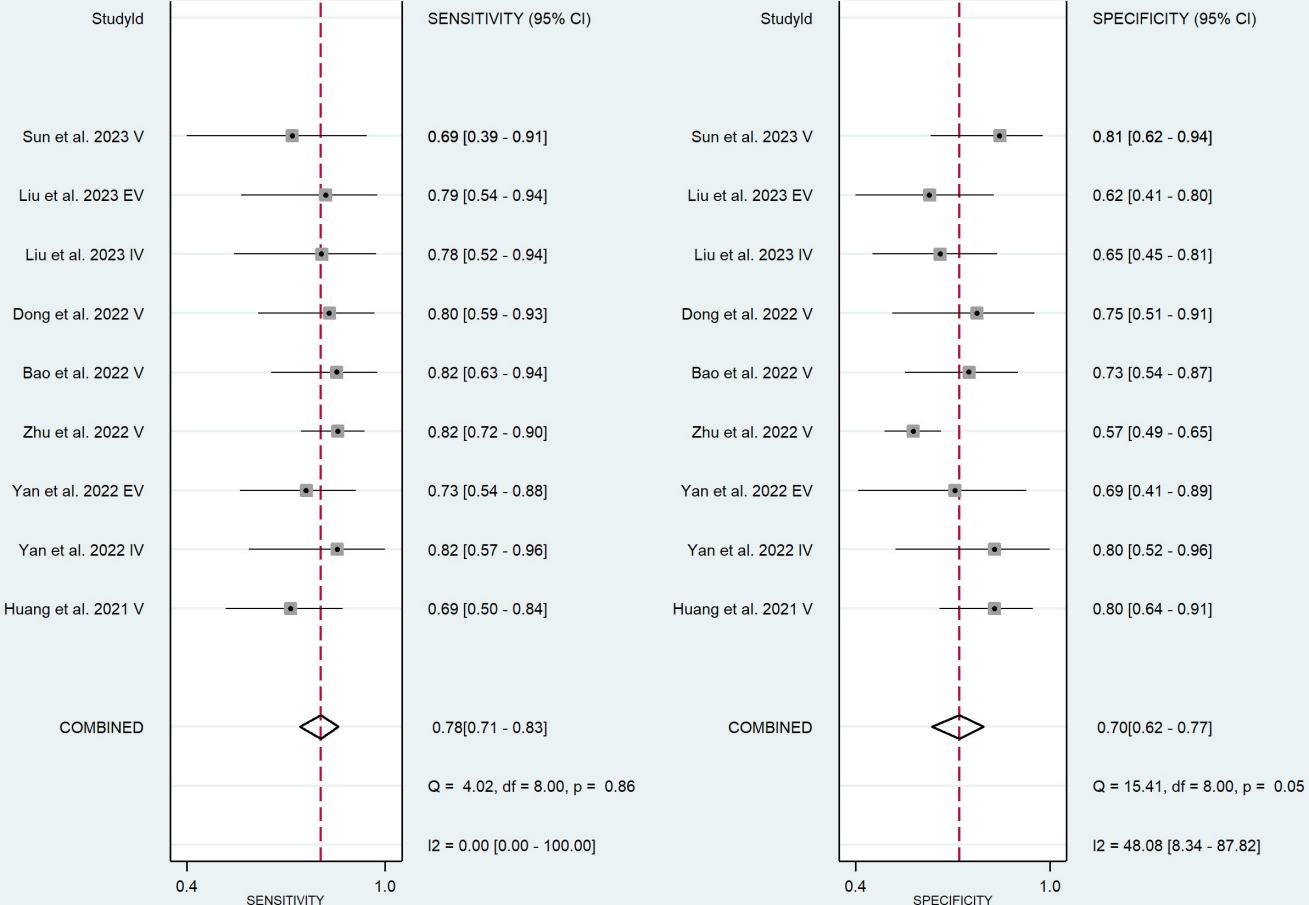
Coupled forest plot of the diagnostic performance in validation cohorts.

Nine validation cohorts consisting of 256 lesions with high and 366 lesions with low Ki-67 were included for meta-analysis. The pooled sensitivity, specificity, DOR, PLR, NLR, and AUC with 95% CI were 0.78 (0.71-0.83), 0.70 (0.62-0.77), 8 (5-12), 2.6 (2.0-3.3), 0.32 (0.25-0.41), and 0.81 (0.77-0.84), respectively. The coupled forest plot showing sensitivity, specificity, and heterogeneity indicators (I^2^ and Cochran’s Q) for validation cohorts is depicted in **Figure 5**. In addition, the SROC with pooled AUC value of the validation cohorts is illustrated in **Figure 4B**.

#### 3.4.3 Heterogeneity assessment

##### 3.4.3.1 Training cohorts’ heterogeneity

The Higgins’ I^2^ value and p-value of Cochran’s Q for sensitivity and specificity were 74.20% (p-value=0.00) and 88.86% (p-value=0.00), respectively, indicating a high heterogeneity among training cohorts. The Spearman’s correlation coefficient (r) was -0.402 (p-value=0.249), indicating lack of threshold effect as the possible cause of heterogeneity.

##### 3.4.3.2 Validation cohorts’ heterogeneity

The Higgins’ I^2^ value and p-value of Cochran’s Q for sensitivity and specificity were 0.00% (p-value=0.86) and 48.08% (p-value=0.05), respectively, indicating a low heterogeneity among validation cohorts. The Spearman’s correlation coefficient (r) was +0.383 (p-value=0.275), indicating lack of threshold effect as the possible cause of heterogeneity.

#### 3.4.4 Heterogeneity exploration using meta-regression

##### 3.4.4.1 Training cohorts

Meta-regression was performed to rule out the possible source of heterogeneity in training cohorts. Among all covariates that were considered for subgroup analysis, only using contrast-enhanced CT scan images (p-value=0.03), PyRadiomics for feature extraction (p-value=0.00), and Ki-67 cut-off of 14% (p-value=0.00) contributed to the inter-study heterogeneity.

##### 3.4.4.2 Validation cohorts

Only using 3D Slicer for feature extraction in validation cohorts contributed to inter-study heterogeneity (p-value=0.04).

#### 3.4.5 Subgroup analysis

##### 3.4.5.1 Training cohorts

In training cohorts (**Table 3**), the sensitivity of studies with a sample size smaller than 150, non-contrast-enhanced CT, logistic regression for modeling, and PyRadiomics for feature extraction was significantly higher (p-value< 0.01). In addition, the sensitivity of studies that used biopsy for tissue obtaining and LASSO for feature reduction was higher but not statistically significant (0.05<p-value< 0.10). The specificity of studies with a sample size smaller than 150, used PyRadiomics for feature extraction or used semiautomatic segmentation was significantly higher (p-value< 0.05).

**Table 3:** Subgroup analysis and meta-regression results in training cohorts.

Covariates		N	Sensitivity	P_SEN_	Specificity	P_SPEC_	Joint model		
							Likelihood ratio chi^2^	I^2^	P value
Sample size	<150	7	0.80 [0.74 - 0.86]	0.00	0.84 [0.76 - 0.92]	0.03	1.84	0	0.40
	>150	3	0.74 [0.66 - 0.82]		0.73 [0.57 - 0.89]				
Publication year	After 2020	8	0.80 [0.74 - 0.86]	0.22	0.83 [0.75 - 0.91]	0.96	2.09	2	0.35
	Before 2020	2	0.71 [0.59 - 0.82]		0.72 [0.50 - 0.93]				
Segmentation	Manual	8	0.78 [0.72 - 0.84]	0.14	0.79 [0.69 - 0.90]	0.01	4.82	43	0.09
	Semiautomatic	2	0.77 [0.63 - 0.91]		0.91 [0.80 - 1.00]				
Contrast Enhanced CT	Yes	3	0.74 [0.61 - 0.86]	0.01	0.87 [0.77 - 0.97]	0.56	7.36	**68**	**0.03**
	No	7	0.80 [0.73 - 0.86]		0.78 [0.69 - 0.87]				
Reference standard	Surgery	7	0.77 [0.70 - 0.84]	0.08	0.82 [0.71 - 0.93]	0.53	1.23	0	0.54
	Biopsy	3	0.82 [0.72 - 0.91]		0.83 [0.66 - 0.99]				
Ki-67 cut-off	5%	2	0.81 [0.70 - 0.91]	0.01	0.82 [0.66 - 0.98]	0.30	0.26	0	0.88
	Other	8	0.77 [0.71 - 0.84]		0.80 [0.71 - 0.89]				
	10%	2	0.75 [0.64 - 0.86]	0.01	0.70 [0.52 - 0.89]	0.04	2.05	0	0.36
	Other	8	0.79 [0.73 - 0.85]		0.83 [0.75 - 0.90]				
	14%	9	0.77 [0.71 - 0.82]	0.73	0.78 [0.71 - 0.84]	0.13	4.77	**83**	**0.00**
	Other	1	0.89 [0.76 - 1.00]		0.95 [0.89 - 1.00]				
	40%	4	0.76 [0.66 - 0.86]	0.01	0.84 [0.71 - 0.97]	0.27	2.21	0	0.33
	Other	6	0.79 [0.72 - 0.86]		0.81 [0.69 - 0.93]				
	50%	1	0.73 [0.57 - 0.88]	0.05	0.67 [0.37 - 0.96]	0.16	1.25	0	0.54
	Other	9	0.79 [0.73 - 0.85]		0.82 [0.74 - 0.89]				
Modeling Algorithm	LR	7	0.78 [0.72 - 0.85]	0.02	0.81 [0.72 - 0.90]	0.25	0.01	0	1.00
	Others	3	0.77 [0.68 - 0.88]		0.80 [0.66 - 0.94]				
Feature Extraction Software	AK	7	0.76 [0.70 - 0.83]	0.02	0.81 [0.72 - 0.91]	0.18	1.92	25	0.38
	Others	3	0.81 [0.74 - 0.89]		0.79 [0.63 - 0.94]				
Feature Extraction Software	3D Slicer	2	0.70 [0.61 - 0.80]	0.00	0.71 [0.51 - 0.91]	0.07	3.21	39	0.20
	Others	8	0.80 [0.75 - 0.85]		0.84 [0.75 - 0.90]				
Feature Extraction Software	PyRadiomics	2	0.89 [0.80 - 0.98]	0.00	0.96 [0.93 - 1.00]	0.00	18.98	**91**	**0.00**
	Others	8	0.75 [0.71 - 0.79]		0.73 [0.69 - 0.78]				
Feature Selection	LASSO	6	0.80 [0.74 - 0.87]	0.06	0.81 [0.71 - 0.90]	0.23	2.92	44	0.17
	Others	4	0.75 [0.66 - 0.84]		0.81 [0.69 - 0.93]				

Bolded values considered significant.

##### 3.4.5.2 Validation cohorts

In validation cohorts (**Table 4**), the sensitivity and specificity of studies with a sample size smaller than 150 were significantly higher (p-value< 0.01). In addition, the sensitivity of studies that used surgery for tissue obtaining and 3D Slicer for feature extraction was higher but not statistically significant (0.05<p-value< 0.10). In addition, studies that used semiautomatic segmentation had a higher specificity but were not statistically significant (0.05<p-value< 0.10).

**Table 4:** Subgroup analysis and meta-regression results in validation cohorts.

Covariates		N	Sensitivity	P_SEN_	Specificity	P_SPEC_	Joint model		
							Likelihood ratio chi2	I^2^	P value
Sample size	<150	7	0.78 [0.71 - 0.85]	0.01	0.72 [0.64 - 0.80]	0.04	0.73	0	0.69
	>150	2	0.77 [0.68 - 0.87]		0.66 [0.54 - 0.78]				
Segmentation	Manual	8	0.78 [0.73 - 0.84]	1.00	0.68 [0.61 - 0.76]	0.08	1.86	0	0.39
	Semiautomatic	1	0.69 [0.44 - 0.95]		0.82 [0.65 - 0.98]				
Contrast Enhanced CT	Yes	1	0.69 [0.44 - 0.95]	0.23	0.82 [0.65 - 0.98]	0.83	1.86	0	0.39
	No	8	0.78 [0.73 - 0.84]		0.68 [0.61 - 0.76]				
Reference standard	Surgury	6	0.78 [0.72 - 0.84]	0.09	0.70 [0.61 - 0.79]	0.18	0.12	0	0.94
	Biopsy	3	0.76 [0.64 - 0.88]		0.69 [0.57 - 0.82]				
Ki-67 cut-off	5%	3	0.73 [0.64 - 0.83]	0.00	0.77 [0.66 - 0.88]	0.55	2.69	26	0.26
	Other	6	0.80 [0.74 - 0.86]		0.66 [0.58 - 0.74]				
	10%	2	0.82 [0.75 - 0.90]	0.05	0.60 [0.53 - 0.67]	0.00	3.97	50	0.14
	Other	7	0.75 [0.69 - 0.82]		0.73 [0.67 - 0.80]				
	14%	1	0.80 [0.64 - 0.97]	0.41	0.75 [0.53 - 0.97]	0.85	0.37	0	0.83
	Other	8	0.77 [0.71 - 0.83]		0.70 [0.62 - 0.77]				
	40%	3	0.76 [0.64 - 0.88]	0.04	0.69 [0.57 - 0.82]	0.14	0.12	0	0.94
	Other	6	0.78 [0.72 - 0.84]		0.70 [0.61 - 0.79]				
Modeling Algorithm	LR	7	0.77 [0.71 - 0.84]	0.01	0.72 [0.64 - 0.79]	0.46	0.92	0	0.63
	Others	2	0.79 [0.69 - 0.89]		0.64 [0.51 - 0.77]				
Feature Extraction Software	AK	5	0.75 [0.67 - 0.83]	0.00	0.71 [0.61 - 0.81]	0.18	0.70	0	0.71
	Others	4	0.80 [0.73 - 0.87]		0.69 [0.58 - 0.79]				
Feature Extraction Software	3D Slicer	1	0.82 [0.74 - 0.91]	0.07	0.57 [0.49 - 0.65]	0.00	6.26	**68**	**0.04**
	Others	8	0.76 [0.70 - 0.83]		0.73 [0.67 - 0.79]				
Feature Extraction Software	PyRadiomics	1	0.80 [0.64 - 0.97]	0.41	0.75 [0.53 - 0.97]	0.85	0.37	0	0.83
	Others	8	0.77 [0.71 - 0.83]		0.70 [0.62 - 0.77]				
Feature Selection	LASSO	8	0.77 [0.71 - 0.83]	0.41	0.70 [0.62 - 0.77]	0.85	0.37	0	0.83
	Others	1	0.80 [0.64 - 0.97]		0.75 [0.53 - 0.97]				

Bolded values considered significant.

#### 3.4.6 Publication bias

**Figure 6:**
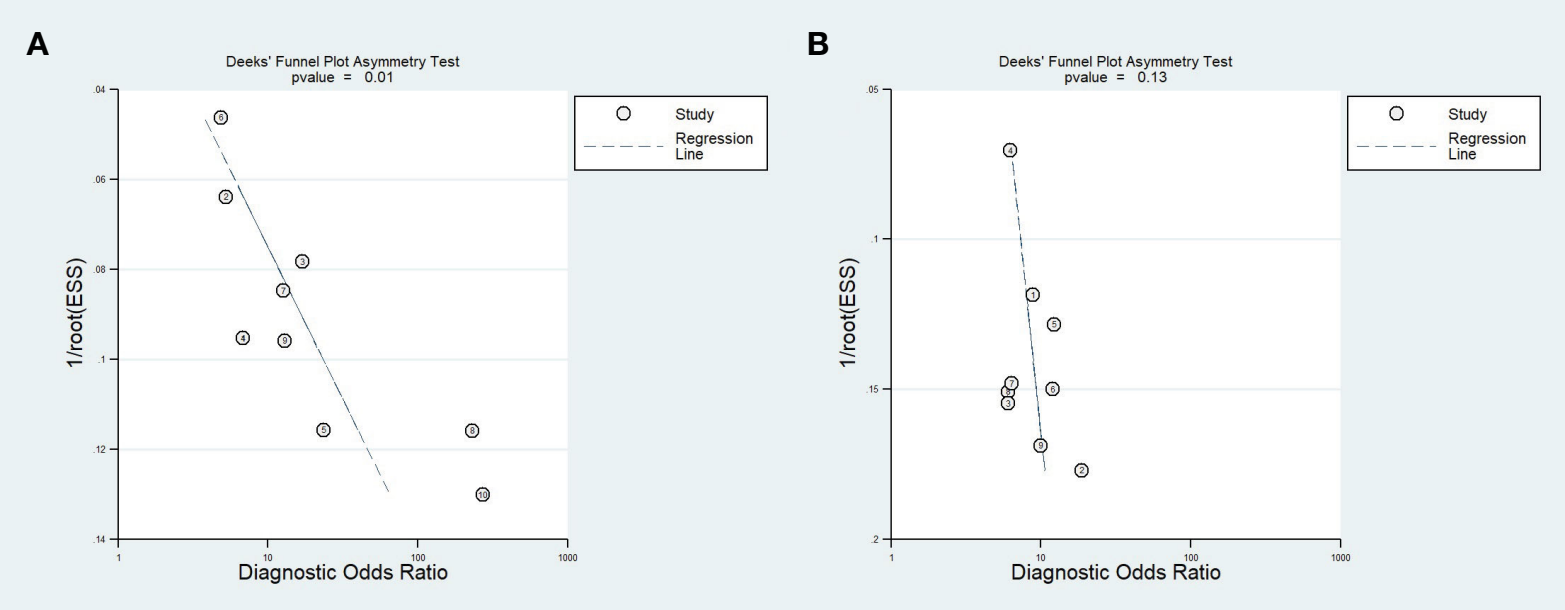
Deeks’ funnel plots for training **(A)** and validation **(B)** cohorts.

We found publication bias in training cohorts based on Deeks’ asymmetry test (p-value=0.01). However, in validation cohorts, publication bias was not significant (p-value <0.13). Deeks’ funnel plots are shown in **Figure 6**. Publication bias was observed exclusively in training cohorts, which typically exhibit higher diagnostic performance compared to validation cohorts. This occurrence, where training cohorts tend to produce more positive results than negative ones, is a prevalent cause of potential publication bias. Various factors, including the inclination to publish novel or statistically significant findings, can contribute to the emergence of this bias.

#### 3.4.7 Sensitivity analysis

To find the possible source of publication bias in training cohorts, we eliminated each study from the analysis and pooled the remaining studies to re-evaluate Deeks’ p-value (**Table 5**). It was found that after eliminating the results by Sun et al., Zhe et al., and Gu et al., the p-value increased; however, it still was <0.05. When removing studies two by two, the Deeks’ p-value of remaining results significantly increased, and this increase reached its maximum after the removal of three studies together (p-value=0.22). Therefore, we concluded that these studies might have contributed to the publication bias. It should be noted that overall results, even with removing these three studies, did not change significantly (**Table 5**), suggesting that the results are consistent.

**Table 5:** Results of the sensitivity analysis.

Name of removed study	Deeks’ test P-value	SEN	SPEC	PLR	NLR	DOR	AUC
**Zhou et al. (** 30 **),**	0.01	0.79	0.83	4.7	0.25	19	0.86
**Gu et al. (** 38 **),**	0.03	0.79	0.84	4.9	0.25	20	0.86
**Huang et al. (** 29 **),**	0.02	0.79	0.82	4.5	0.26	17	0.86
**Fu et al. (** 33 **),**	0.01	0.79	0.83	4.7	0.25	19	0.86
**Yan et al. (** 37 **),**	0.02	0.77	0.83	4.6	0.27	17	0.84
**Zhu et al. (** 34 **),**	0.03	0.79	0.84	5.0	0.25	20	0.86
**Bao et al. (** 36 **),**	0.02	0.78	0.84	4.8	0.26	18	0.85
**Dong et al. (** 32 **),**	0.01	0.77	0.78	3.4	0.30	11	0.83
**Liu et al. (** 31 **),**	0.02	0.78	0.82	4.3	0.27	16	0.85
**Sun et al. (** 35 **),**	0.05	0.77	0.79	3.6	0.29	12	0.83
**Gu AND Zhu**	0.08	0.80	0.86	5.7	0.23	25	0.88
**Gu AND Sun**	0.08	0.78	0.80	4.0	0.28	14	0.85
**Zhu AND Sun**	0.10	0.78	0.80	4.0	0.27	15	0.85
**Zhu AND Sun AND Gu**	0.22	0.79	0.82	4.5	0.26	18	0.85

#### 3.4.8 Leave-one-out analysis

Leave-one-out analysis was performed to investigate the robustness of the results by removing each study one by one. In the included training cohorts, it was observed that upon excluding the cohorts by Sun et al. or Dong et al., there was a slight decrease in the values of specificity, PLR, and DOR. This suggests that these cohorts exhibited slightly higher diagnostic performance compared to other studies (**Supplementary Figures S1-3**). However, no significant changes were noted in the sensitivity, specificity, PLR, NLR, or DOR values upon the removal of each validation cohort (**Supplementary Figures S4-6**). Taken together, no concern existed regarding the robustness of the results.

## 4 Discussion

The present meta-analysis showed that CT-based radiomics have an excellent diagnostic performance for predicting Ki-67 expression with pooled AUCs > 0.80 in both training and validation cohorts of ten studies. In addition, the quality of the included studies was evaluated using two different tools, including QUADAS-2 and RQS, indicating that the included studies have an acceptable quality. The interpretation of results in a meta-analysis is significantly impacted by the quality of the included articles. In the realm of radiomics research and the application of the RQS, the overall reliability of meta-analysis findings relies on the methodological rigor and thoroughness of each individual study. If the articles included in the analysis have low RQS scores, indicating inadequate methodological quality and presentation, it introduces a risk of bias and reduces confidence in the pooled results. Additionally, variations in study design, reporting, and validation practices among low-quality studies can introduce heterogeneity, complicating the integration of data and potentially leading to less robust conclusions. Thus, a careful evaluation of the quality of individual articles, facilitated by tools like RQS, becomes crucial to ensure the accuracy and applicability of meta-analytic outcomes in the field of radiomics and beyond. In a recently published meta-analysis on the diagnostic performance of MRI-based radiomics for predicting Ki-67 in breast cancer (24), the mean RQS of the included studies was around 6, significantly lower compared to our study. This suggests that the quality of the articles included in our meta-analysis was higher.

A meta-analysis encompassing 15 studies and 1931 patients demonstrated the prognostic significance of Ki-67 in stage I NSCLC. The analysis revealed that a high Ki-67 labeling index (LI) in stage I NSCLC is predictive of poorer OS and DFS. Furthermore, the study explored the prognostic impact, specifically in stage I adenocarcinoma, providing novel insights. Despite acknowledged limitations, such as reliance on pooled data and potential publication bias, the meta-analysis recommends Ki-67 as a routine biomarker in stage I NSCLC. It suggests that patients with high Ki-67 LI may benefit from adjuvant therapy (10). These data highlight the prominent role of Ki-67 in the prognosis of lung cancer patients. The limitations of lung cancer biopsy include poor discriminatory capability of imaging, late diagnosis, variability in diagnostic pathways, and potential complications from biopsy procedures (39). Radiomics, a field focused on extracting and analyzing quantitative features from medical images, offers a promising solution to these challenges. By providing a more detailed and comprehensive characterization of lesions, radiomics enhances the discriminatory capability of imaging, aids in early detection, and enables risk stratification. Additionally, it supports personalized medicine by identifying tumor heterogeneity and potential molecular targets, potentially reducing the need for invasive biopsies. Radiomics also facilitates monitoring treatment response, allowing clinicians to assess therapy effectiveness and make timely adjustments to treatment plans (40–42). Integrating radiomics into current prognostic workflows for predicting Ki-67 expression in lung cancer involves incorporating radiomic features extracted from imaging data, such as CT scans, into existing prognostic models. This process requires the development of algorithms that capture intricate patterns related to Ki-67 expression, with subsequent validation against established indicators and clinical outcomes (43, 44). Collaboration between radiologists, oncologists, and data scientists is crucial for optimization, standardization, and the establishment of protocols. Education programs for healthcare professionals ensure proper interpretation of radiomic data, and continuous refinement through research and clinical feedback contributes to the ongoing improvement of these models. The successful integration of radiomics necessitates a multidisciplinary approach, technological standardization, and collaborative efforts across healthcare settings (45).

CT-based radiomics emerges as an efficient tool for predicting Ki-67 expression in lung cancer, particularly in NSCLC, providing several advantages. The ease of acquisition and non-invasiveness of CT scans allows for early Ki-67 expression prediction. The integration of radiomics-based analyses with radiologist assessment proves beneficial in clinical decision-making for NSCLC patients. This approach has already been investigated for predicting EGFR mutation status, facilitating the identification of patients suitable for targeted therapies. CT radiomics-based models present distinct advantages over conventional methods by leveraging existing imaging data, eliminating the need for invasive procedures, and minimizing patient discomfort and risk. Routine CT scans yield readily available data for radiomics-based models, enabling a comprehensive assessment of tumor characteristics throughout the entire tumor volume. Advanced imaging analysis techniques quantify features, enhancing the precision of predicting genetic mutations. Serial CT scans enable longitudinal monitoring of tumor characteristics, allowing for the evaluation of treatment response, disease progression, and monitoring emerging genetic mutations over time. While CT radiomics-based models should not replace confirmatory molecular testing methods, they significantly contribute to noninvasive and comprehensive assessments in lung cancer management (46).

In this meta-analysis, we observed a significant heterogeneity in training cohorts, which made meta-regression necessary. First, we observed that contrast-enhanced CT-scan images contributes to inter-study heterogeneity. This finding was justifiable as inconsistencies and differences can occur in different process of enhanced CT-scan acquisition due to various reasons such as temporal imaging and lesion enhancement patterns. Second, we found that using PyRadiomcis for feature extraction contributed to interstudy heterogeneity. Utilizing diverse software tools for radiomics feature extraction introduces interstudy heterogeneity, primarily stemming from algorithmic dissimilarities, variations in parameter configurations, discrepancies in segmentation methods, differences in image preprocessing approaches, disparities in normalization and scaling procedures, diverse criteria for feature selection, and potential inconsistencies in software updates and versions. These distinctions in radiomic processes yield distinct sets of features, complicating direct comparisons between studies (47). The superiority of PyRadiomics compared to other feature extraction software has also been mentioned in a previous meta-analysis (48). Lastly, using a Ki-67 cut-off of 14% could also cause interstudy heterogeneity. Different cut-off values for Ki-67 expression were used in the included studies, causing a significant heterogeneity.

Our subgroup analyses have revealed common findings in validation and training cohorts. Firstly, studies with sample sizes smaller than 150 had a significantly higher diagnostic performance. The observed phenomenon of significantly higher diagnostic performance in studies with sample sizes smaller than 150 could be attributed to various factors. These include the sensitivity of smaller sample sizes to outliers, increased heterogeneity in smaller studies, a potential publication bias that favors the reporting of positive results in smaller studies, a more substantial impact of random variation due to limited sample size, specific clinical contexts where certain diagnostic tests perform better in smaller populations, and interactions between the characteristics of the studied subgroups and the sample size. Each of these factors may contribute to or influence the apparent difference in diagnostic performance observed in the subgroup analysis. Second, we observed that semi-automatic segmentation may increase the specificity of the results. This method allows for accurate delineation of the ROI, enabling correction of errors, customization for complex structures, and adaptability to anatomical variability.

In our meta-analysis, we also observed a significant publication bias for training cohorts based on Deeks’ test. Publication bias happens when studies with positive or statistically significant results are more likely to be published, while studies with null or negative findings are less likely to be published. We identified three studies as the possible source of publication bias. However, eliminating these studies one by one or even together could not change the overall diagnostic performance of the radiomics approach for the prediction of Ki-67 in lung cancer, indicating that the results were consistent. Publication bias occurs when research findings are selectively published based on their nature and direction, often leading to an overemphasis on positive or statistically significant results. To mitigate publication bias, researchers can pre-register studies, encouraging the publication of negative results, and promoting systematic reviews that include unpublished studies. Journals can adopt policies prioritizing study design over results, and open access to data can facilitate result verification. Transparent reporting guidelines, publication of study protocols, and rigorous editorial and peer review processes further contribute to reducing the impact of publication bias, ensuring a more balanced representation of scientific evidence (49, 50).

## 5 Limitations

Several limitations in this meta-analysis warrant consideration:

1. Lack of Validation Cohorts: A notable limitation is the absence of validation cohorts in several studies, necessitating the pooling of training and validation cohorts separately, which may impact the generalizability of the findings.
2. Retrospective Study Design: The retrospective nature of all included studies introduces inherent biases and limits the establishment of causal relationships, emphasizing the need for prospective investigations to validate the observed associations.
3. Geographic Bias: The exclusive focus on studies conducted in China introduces regional bias, potentially limiting the generalizability of findings to a broader, more diverse population.
4. Limited Segmentation Information: While all studies utilized 3D segmentation, the absence of information on 2D segmentation performance for predicting Ki-67 in lung cancer underscores a potential gap in understanding the comparative effectiveness of these methods.
5. Scarcity of Automatic Segmentation: The limited use of automatic or semiautomatic segmentation in only two studies emphasizes the necessity for further research to explore the performance and advantages of automated segmentation methods.
6. Absence of Deep Learning Approaches: The exclusion of deep learning-based radiomics methods in all studies underscores a current gap in exploring the potential benefits of advanced machine learning techniques, which could enhance predictive accuracy (51).
7. Variability in Ki-67 Expression Cutoffs: The inconsistency in defining lesions as positive for Ki-67 expression due to different cutoff points across studies poses a challenge to standardization, suggesting the need for authors to test multiple cutoff points in their investigations.
8. Publication Bias Concerns: The identification of publication bias raises awareness of a potential inclination toward reporting positive results. Encouraging journals and authors to publish negative results can help address this bias and provide a more comprehensive understanding of the predictive capabilities of CT-based radiomics for Ki-67 expression in lung cancer.

## 6 Conclusion

In conclusion, this meta-analysis of 10 retrospective studies investigating CT-based radiomics for predicting Ki-67 expression in lung cancer demonstrates promising diagnostic performance, indicating the potential clinical utility of radiomic features. These findings collectively highlight the potential of radiomics in noninvasive prediction of Ki-67 expression, emphasizing the importance of cautious interpretation and the need for further research to address methodological heterogeneity and potential biases.

## Data availability statement

The original contributions presented in the study are included in the article/**Supplementary Material**. Further inquiries can be directed to the corresponding author.

## Author contributions

XL: Writing – original draft, Writing – review & editing. RZ: Writing – original draft, Writing – review & editing. JZ: Writing – original draft, Writing – review & editing. JH: Writing – original draft, Writing – review & editing. WL: Writing – original draft, Writing – review & editing. ZJ: Writing – original draft, Writing – review & editing. QL: Writing – review & editing.
