# Supplementary material for: CT-based radiomics for predicting Ki-67 expression in lung cancer: a systematic review and meta-analysis
Source: Front Oncol. 2024 Feb 7;14:1329801. doi: 10.3389/fonc.2024.1329801 (PMC10879429; doi:10.3389/fonc.2024.1329801)
Supplement: Supplementary file 1 [file DataSheet_1.docx]

Table 1- Embase

| Set | Query | Records |
| --- | --- | --- |
| #1 | **ki67 OR 'ki 67'/exp OR 'ki 67' OR 'mitotic index'/exp OR 'mitotic index' OR (mitotic AND ('index'/exp OR index)) OR 'proliferation index'/exp OR 'proliferation index' OR (('proliferation'/exp OR proliferation) AND ('index'/exp OR index)) OR (mib AND ('1'/exp OR 1)) OR 'mib 1'/exp OR 'mib 1' OR 'mitosis index'/exp OR 'mitosis index' OR (('mitosis'/exp OR mitosis) AND ('index'/exp OR index))** | 186,322 |
| #2 | 'radiomics'/exp OR 'radiomics' OR 'machine learning'/exp OR 'machine learning' OR 'artificial intelligence'/exp OR 'artificial intelligence' OR 'deep learning'/exp OR 'deep learning' OR 'neural network'/exp OR 'neural network' OR 'artificial neural network'/exp OR 'artificial neural network' OR 'radiomics model'/exp OR 'radiomics model' OR 'radiogenomics'/exp OR 'radiogenomics' OR 'radiomics nomogram' OR 'computer assisted diagnosis'/exp OR 'computer assisted diagnosis' OR 'texture analysis'/exp OR 'texture analysis' OR 'feature learning (machine learning)'/exp OR 'feature learning (machine learning)' OR 'feature extraction'/exp OR 'feature extraction' OR 'detection algorithm'/exp OR 'detection algorithm' OR 'imaging algorithm'/exp OR 'imaging algorithm' OR 'image processing'/exp OR 'image processing' | 2,039,362 |
| #4 | 'lung cancer' OR 'non small cell lung cancer' OR 'lung tumor' OR 'lung adenocarcinoma cell line' OR 'lung adenocarcinoma' OR 'nsclc' | 508,491 |
| Combined set | **#1 AND #2 AND #3 AND #4** | 1377 |

Table 2-PubMed

| Set | Query | Records |
| --- | --- | --- |
| #1 | **(Ki-67) OR ("KI 67") OR (KI67) OR (ki67) OR (ki-67) OR (mitotic index) OR (proliferation index) OR (MIB 1) OR (MIB-1) OR (mitosis index)** | 73,227 |
| #2 | **((Radiomics) OR (machine learning) OR (machine AND learning) OR (deep learning) OR (deep AND learning) OR (neural network) OR (neural AND network) OR (radiomics nomogram) OR (Algorithms) OR (Artificial Intelligence) OR (Artificial AND Intelligence) OR (computer-assisted diagnosis) OR (texture analysis) OR (feature AND extract*) OR (image AND process*) OR (texture AND analysis) OR (radiogenomic*))** | 1,229,033 |
| #3 | **(lung cancer) OR "NSCLC" OR "lung tumor" OR "lung neoplasms"[Mesh] OR "lung neoplasm*"[Title/Abstract] OR "lung tumor*"[Title/Abstract] OR "lung cancer*"[Title/Abstract] OR "lung carcinoma*"[Title/Abstract] OR "lung neoplasm*"[Title/Abstract] OR "lung carcinoma*"[Title/Abstract]** | 434,783 |
| Combined set | **#1 AND #2 AND #3** | 114 |

Table 3- Web of Science

| Set | Query | Records |
| --- | --- | --- |
| #1 | ALL=("KI 67") OR ALL=("KI67") OR ALL=("ki67") OR ALL=("ki-67") OR ALL=("mitotic index") OR ALL=("proliferation index") OR ALL=("MIB 1") OR ALL=("MIB-1") OR ALL=("mitosis index") | 53,405 |
| #2 | (((((((ALL=(Radiomics)) OR ALL=(machine learning)) OR ALL=(deep learning)) OR ALL=(neural network)) OR ALL=(radiomics nomogram)) OR ALL=(texture analysis)) OR ALL=(feature extraction)) OR ALL=(radiogenomics) | 894,055 |
| #3 | ALL=(lung cancer) OR ALL=(lung Neoplasm) OR ALL=(lung tumor) OR ALL=(NSCLC) OR ALL=(Lung Adenocarcinoma) | 556,473 |
| Combined set | **#1 AND #2 AND #3 AND** | 41 |


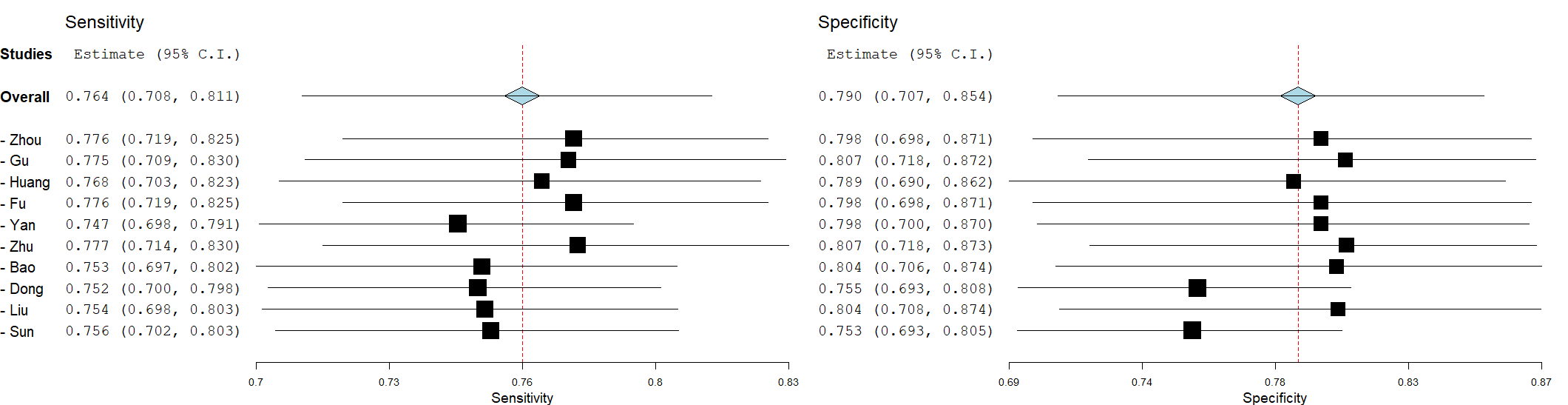


**Figure S1**- Leave-one-out analysis of the training cohorts showed that removing Dong and Sun cohorts could lower the specificity value.


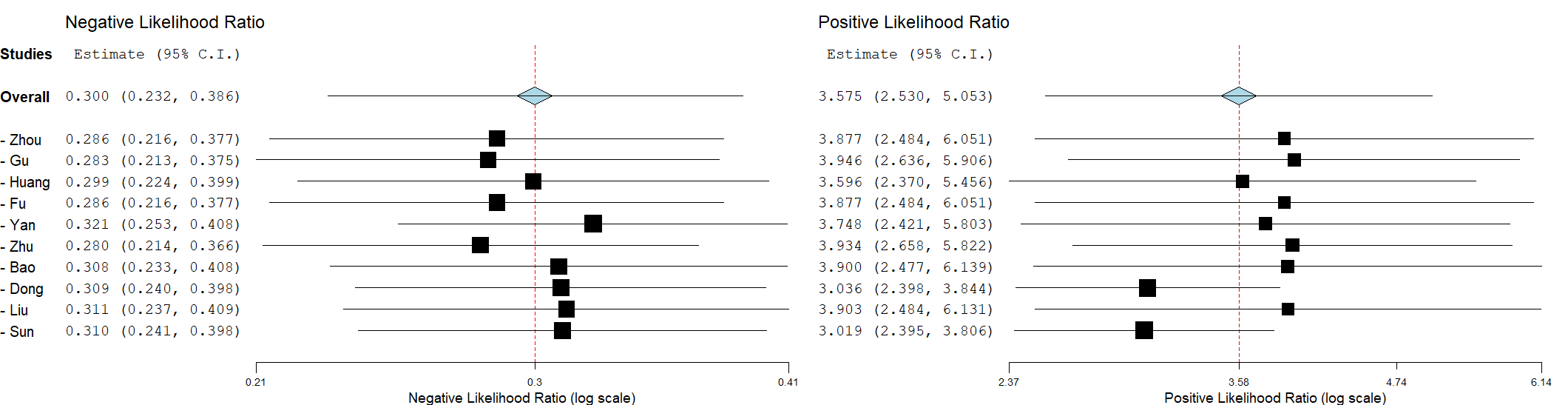


**Figure S2-** Leave-one-out analysis of the training cohorts showed that removing Dong and Sun cohorts could lower the PLR value but not NLR.


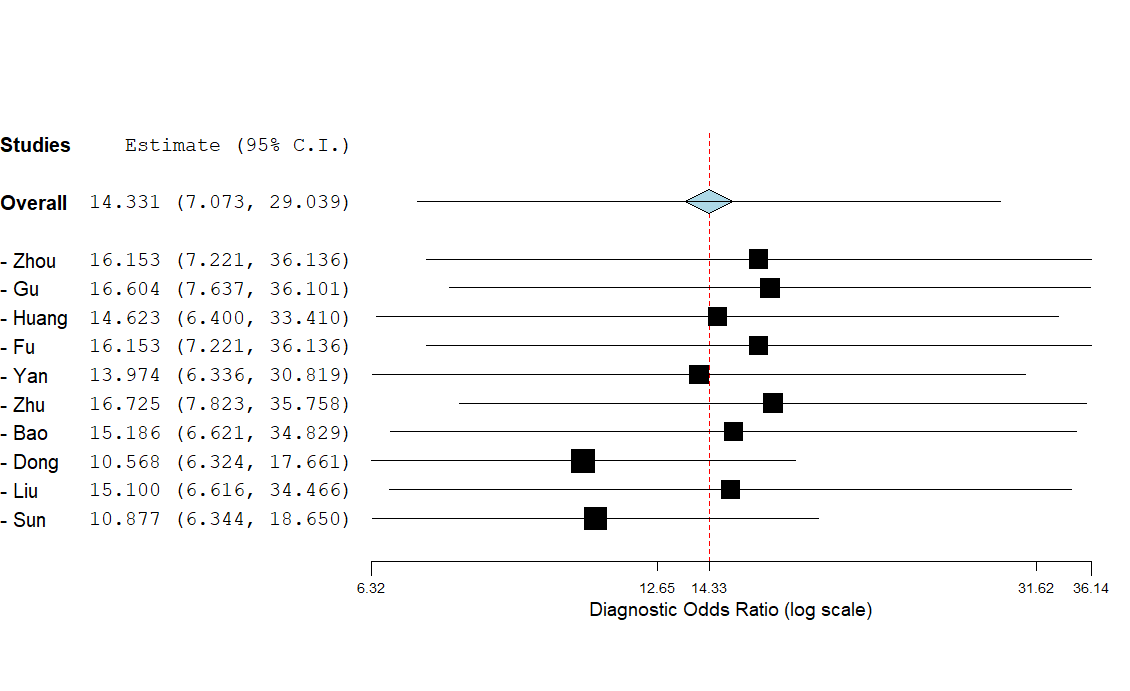


**Figure S3**- Leave-one-out analysis of the training cohorts showed that removing Dong and Sun cohorts could lower the DOR value.


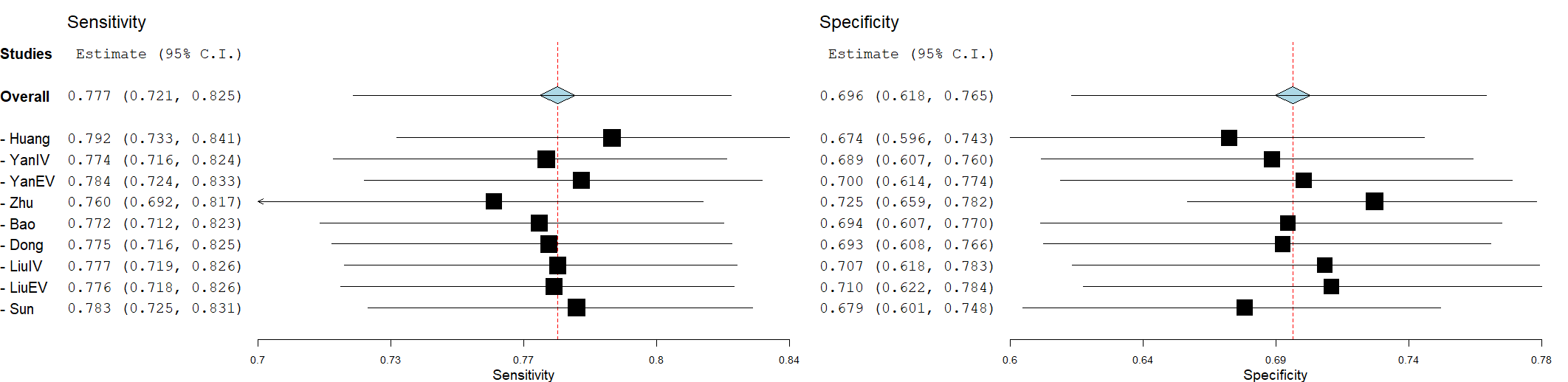


Figure S4- Leave-one-out analysis of the validation cohorts did not show significant changes for sensitivity and specificity.


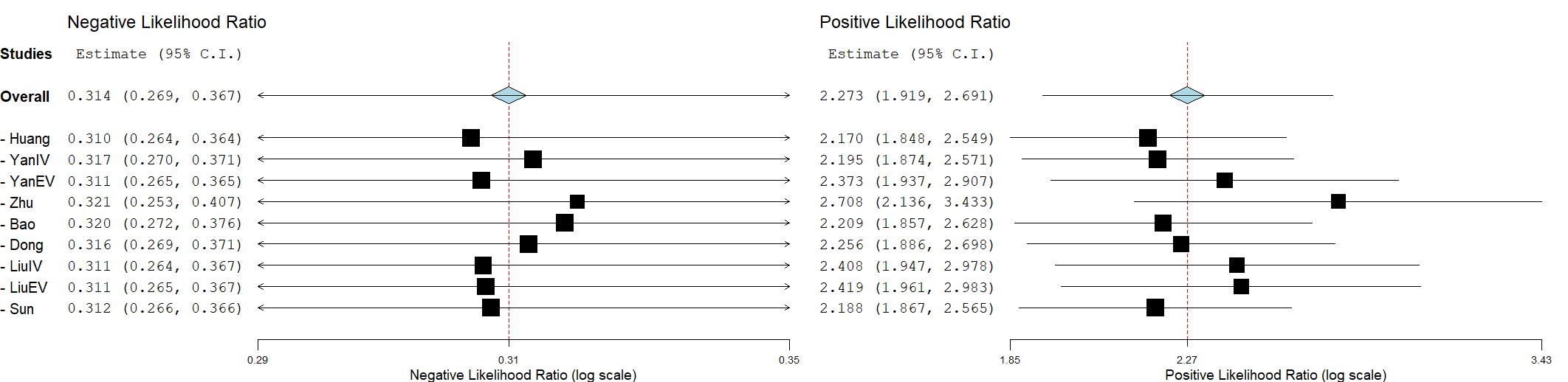


**Figure S5-** Leave-one-out analysis of the validation cohorts did not show significant changes for NLR and PLR.


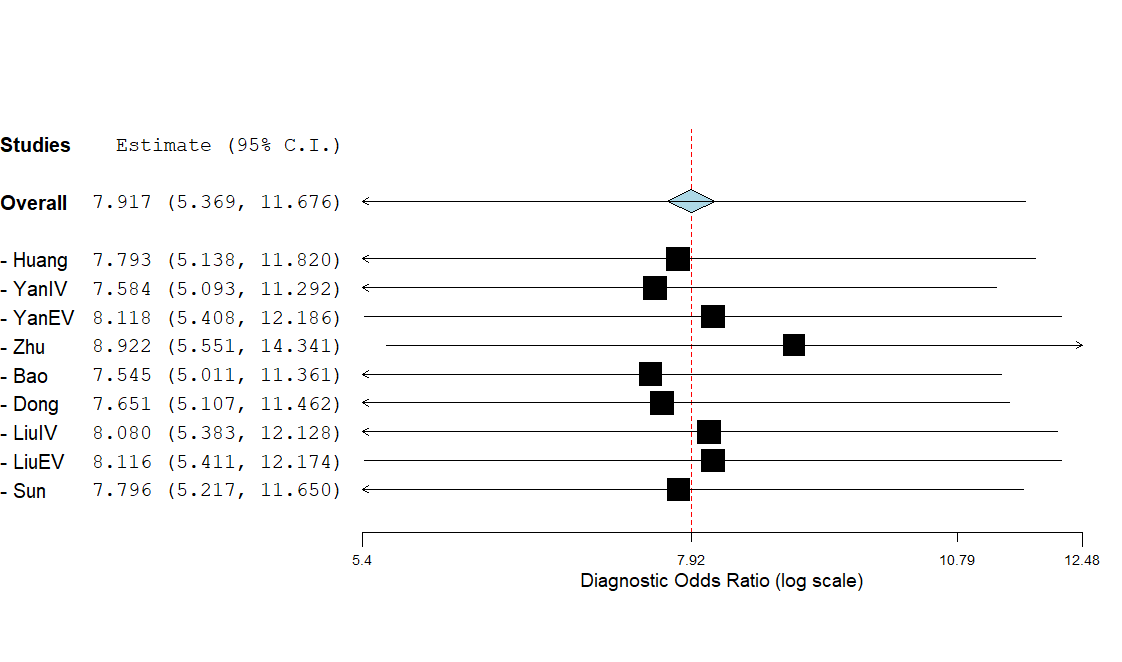


**Figure S6**- Leave-one-out analysis of the validation cohorts did not show significant changes for DOR.
